# Integrated multi-omics mapping of the causal landscape of gout across the circulating-tissue axis

**DOI:** 10.3389/fimmu.2026.1776456

**Published:** 2026-02-24

**Authors:** Liang Huang, Jiani Liu, Xiaohui Zheng, Kai Zhang, Yixin Chen, Xiaoling Chen, Siqi Zhang, Shanshan Cai, Li Cai, Yanyan Guo, Peng Zhu, Meng Li

**Affiliations:** 1 Department of Radiology, the Second Affiliated Hospital, University of South China, Hengyang, Hunan, China; 2 Department of Traditional Chinese Internal Medicine, School of Traditional Chinese Medicine, Southern Medical University, Guangzhou, China; 3 Nanjing Hospital of Chinese Medicine, Nanjing University of Chinese Medicine, Nanjing, China; 4 The First School of Clinical Medicine, Southern Medical University, Guangzhou, Guangdong, China; 5 Division of Biomedical and Life Sciences, Faculty of Health and Medicine, Lancaster University, Lancaster, United Kingdom; 6 Department of Nephrology and Rheumatology, The Affiliated Traditional Chinese Medicine Hospital, Guangzhou Medical University, Guangzhou, China; 7 Department of Radiology, Taishan People’s Hospital, Jiangmen, Guangdong, China; 8 Clinical Laboratory, Shenzhen Pingshan District People’s Hospital, Pingshan Hospital, Southern Medical University, Shenzhen, Guangdong, China; 9 Nanfang Hospital, Southern Medical University, Guangzhou, Guangdong, China

**Keywords:** causal biomarkers, colocalization, gout, Mendelian randomization, multi-omics

## Abstract

**Background:**

Gout is a prevalent inflammatory arthropathy driven by monosodium urate crystal deposition, yet the causal relationships between circulating biomarkers and disease susceptibility remain incompletely characterized. Establishing robust causal associations and mapping them to specific effector genes and tissues is essential for identifying mechanistically informed therapeutic targets.

**Methods:**

We conducted a comprehensive multi-omics Mendelian randomization study integrating a meta-analysis of three large-scale gout genome-wide association studies (N = 1,538,494) with genome-wide data for 233 metabolites, 179 lipid species, and 926 plasma proteins. Findings were replicated in an independent cohort (N = 327,457). Summary-data-based Mendelian randomization and Bayesian colocalization (HyPrColoc) were applied to map causal biomarkers to tissue-specific effector genes using expression quantitative trait loci data from kidney, liver, and whole blood. Candidate genes were experimentally validated in monosodium urate-stimulated THP-1 macrophages.

**Results:**

We identified 32 metabolites, one lipid species (TAG 54:3), and two protective plasma proteins (ISLR2, ITIH3) with replicated causal associations with gout. Triglyceride-rich very-low-density lipoprotein particles and circulating isoleucine emerged as prominent risk factors. Multi-tissue transcriptomic mapping prioritized *PRELID1* (kidney), *NIPAL1* (liver), *LMAN2* (whole blood), and *CAD* as high-confidence effector genes with strong colocalization evidence (posterior probability >0.70). Functional validation confirmed concordant transcriptional and translational dysregulation of these genes following inflammatory stimulation.

**Conclusion:**

This integrative analysis establishes a causal framework linking specific lipoprotein subfractions, amino acid metabolism, and novel effector genes to gout pathogenesis, elucidating the systemic metabolic architecture of the disease and identifying potential therapeutic candidates warranting further preclinical investigation before clinical translation.

## Introduction

Gout, a prevalent and complex metabolic disorder, represents one of the most common inflammatory arthropathies affecting millions of individuals worldwide, characterized by acute and recurrent inflammatory episodes triggered by monosodium urate (MSU) crystal deposition in joints and surrounding tissues (1, 2). The pathophysiology of gout is intricately linked to hyperuricemia, elevated serum uric acid (SUA) levels that exceed the solubility threshold of uric acid, leading to MSU crystallization and subsequent immune activation (3, 4). While uric acid is the end product of purine metabolism in humans, dysregulation of complex metabolic pathways involving multiple genetic loci contributes to the disease susceptibility and progression (2, 5, 6).

Recent advances in genomic technologies have substantially improved our understanding of gout genetics. Large-scale genome-wide association studies (GWAS) have identified numerous risk loci including *NLRP3*, *MAP3K11*, *TRIM46*, and *TM7SF2* that encode urate transporters critically involved in renal and intestinal urate handling (2, 7). These discoveries provide a more solid biological foundation for targeted therapies in gout, enabling the development of upstream interventions with fewer side effects and a higher degree of personalization. Despite these advances, the causal relationships between circulating biomarkers and gout development remain incompletely characterized, limiting our ability to translate genetic findings into mechanism-based treatments. Although several small, single-ancestry Mendelian randomization (MR) studies have reported putative causal plasma metabolites, including hexanoylglutamine and phenylacetylcarnitine, among others, these efforts are only an initial step (8, 9). Establishing a causal association between a circulating biomarker and gout is necessary but not sufficient. To ensure the robustness of such findings and to facilitate rational drug discovery, it is essential to map these biomarkers to specific genes and tissues. However, due to limitations in GWAS design and analysis, many studies have not adequately addressed issues such as weak instruments, pleiotropy, and other sources of noise. As a result, the implicated metabolites often lack consistent or reproducible causal effects. In particular, most prior work has largely stopped at using MR to identify putative causal relationships, with insufficient statistical sensitivity analyses and little effort to elucidate and validate the underlying molecular mechanisms. Addressing these gaps constitutes the core objective of our study.

In this study, we performed a comprehensive multi-omics post-GWAS analysis to identify key causal biomarkers and effector genes for gout. To maximize statistical power, we first conducted a large-scale meta-analysis of three of the most recent major gout GWAS datasets. Because proteins serve as the functional executors of genetic information and represent the most proximal molecular layer to disease phenotypes, integrating proteomic data with genomic findings is essential for translating genetic associations into mechanistically actionable targets. We then systematically screened circulating metabolic biomarkers, lipid species, and thousands of plasma proteins from independent consortia. To validate the robustness and generalizability of our findings, we used an additional gout GWAS dataset as an independent replication cohort. Subsequently, we integrated tissue-specific eQTL data from relevant tissues and applied Summary-data-based Mendelian Randomization (SMR) and HyPrColoc to map prioritized biomarkers to their encoding genes and to confirm shared genetic etiology. Finally, to ensure the biological relevance of our computational results, we experimentally validated the expression and function of selected key genes through follow-up functional analyses.

This integrative study aims to generate a high-resolution map of the molecular drivers of gout, providing new insights for precision medicine and therapeutic intervention. By leveraging an integrated multi-omics framework, we seek to dissect the causal hierarchy from genetic variants to circulating biomarkers to disease onset and progression, while identifying novel, mechanistically informed therapeutic targets for gout management.

## Methods

### Study design and data sources

This study employed a multi-stage analytical framework integrating genome-wide association studies, summary-data-based Mendelian randomization, tissue-specific expression quantitative trait loci, and Bayesian colocalization analyses. All analyses utilized publicly available summary-level GWAS data, eliminating the requirement for individual-level participant data and ethical approval.

### Gout GWAS data

Three independent gout GWAS datasets from European ancestry populations were included as the discovery cohort. The first dataset (Major TJ et al., n=642,075) was identified through the GWAS Catalog with identifier GCST90428600 (2). The second dataset (Carss K et al., n=458,440) from non-Finnish European individuals in the UK Biobank was designated GCST90474006 (10). The third dataset (Verma A et al., n=437,979) from the VA Million Veteran Program represented diverse disease-relevant endotypes with identifier GCST90475731 (11). To maximize statistical power and generate robust discovery associations, we conducted a fixed-effects inverse-variance weighted meta-analysis of these three GWAS datasets. The meta-analysis was performed using standard protocols, with heterogeneity assessed via Cochran’s Q test (p-value threshold >0.05 indicating homogeneity). For external validation, we utilized gout GWAS summary statistics from the FinnGen R12 consortium (n=327,457), a national biobank comprising well-characterized Finnish participants with comprehensive health registry data (12).

### Metabolomic, lipidomic, and proteomic GWAS data

Circulating metabolite GWAS summary statistics were obtained from the metabolomic study by Karjalainen et al. (n=136,016 multi-ancestry participants), providing genome-wide associations for 233 metabolic biomarkers (13). The lipidomic GWAS dataset derived from Ottensmann et al. included 179 lipid species measured in 7,174 Finnish individuals from which 495 genetic associations were identified at novel and established loci (14). Plasma proteome GWAS data were obtained from two large-scale proteogenomic studies: the deCODE Genetics consortium proteome study (n=35,559 European participants) (15) and the UK Biobank Plasma Proteomic Project (UKB-PPP, n=54,306 European individuals), both providing cis-pQTL-disease associations (16).

### Two-sample Mendelian randomization analysis

We conducted two-sample MR analysis to evaluate the causal associations between circulating biomarkers and gout risk. The inverse-variance weighted (IVW) method was employed as the primary analytical approach, which assumes balanced horizontal pleiotropy and provides the most precise causal estimates when this assumption is satisfied. For exposures with more than one independent instrumental variable, we utilized either the Wald ratio method (for single SNP instruments) or the random-effect IVW method (for multiple SNPs). To evaluate the robustness of causal estimates and assess sensitivity to pleiotropy violations, we implemented several complementary MR methods including MR-Egger regression, and weighted median (WM) method. Results were considered robust when the direction and significance of causal effects remained consistent across multiple analytical methods.

### Protein dataset integration

To obtain comprehensive proteome-wide coverage while minimizing redundancy, we identified the intersection of proteins present in both the deCODE consortium proteomics dataset and the UK Biobank Pharma Protein Panel (UKB-PPP), resulting in 926 proteins subjected to MR analysis. This integrated approach leverages the strengths of both datasets and ensures robust protein-level associations with gout susceptibility.

### Multiple testing correction strategy

Given the large number of exposures analyzed across three distinct omics categories—233 metabolites, 179 lipid species, and 926 proteins—we applied stringent multiple testing correction using the Benjamini-Hochberg false discovery rate (FDR) procedure within each omics category separately. This stratified FDR correction approach is particularly appropriate for multi-omics discovery studies, as it controls the false discovery rate while maintaining adequate statistical power for each biomarker class independently. For the discovery phase using the gout meta-GWAS, we considered associations statistically significant at an FDR-adjusted threshold of P_FDR < 0.05 within each omics category.

### Discovery and replication validation strategy

To establish robust and reliable associations, we employed a rigorous two-stage discovery and replication design. All biomarkers reaching significance in the discovery phase (gout meta-GWAS, FDR < 0.05) were systematically tested in an independent replication cohort (FinnGen R12 consortium gout GWAS). A finding was considered successfully replicated only when it satisfied both of the following criteria: (1) nominal statistical significance in the replication analysis (P < 0.05), and (2) consistent direction of effect between discovery and replication analyses (identical sign of effect estimate). Associations failing to meet both criteria were excluded from downstream functional analyses. This stringent validation approach ensures that identified causal biomarkers represent genuine, reproducible associations rather than chance findings.

### Heterogeneity and pleiotropy assessment

Heterogeneity among SNP-specific causal estimates was assessed using Cochran’s Q test, with statistical significance indicating potential heterogeneity that might be attributable to pleiotropy or weak instrument bias. We evaluated horizontal pleiotropy—where genetic variants affect the outcome through pathways independent of the primary exposure—using MR-PRESSO (Mendelian Randomization Pleiotropy RESidual Sum and Outlier), which identifies and removes outlier variants that violate MR assumptions. The MR-Egger intercept test was additionally applied to estimate directional pleiotropy, where a statistically significant non-zero intercept (P < 0.05) indicates directional pleiotropy. Results from sensitivity analyses demonstrating substantial heterogeneity or pleiotropy were interpreted with caution and required additional validation through complementary methods.

### Summary-based Mendelian randomization and HEIDI filtering

For biomarkers successfully validated in replication analyses, we performed SMR analysis to identify specific genes whose expression is causally associated with gout risk, utilizing tissue-specific eQTL data from GTEx (kidney, liver, and whole blood). The heterogeneity in dependent instruments (HEIDI) test was applied as a critical quality control filter to distinguish genuine mediation effects from associations driven by linkage disequilibrium or shared variants between eQTL and GWAS summary statistics. We employed a stringent HEIDI test P-value threshold of P_HEIDI > 0.05 to exclude associations with evidence of linkage disequilibrium-driven false positives. Genes passing HEIDI filtering were retained for downstream colocalization analysis and functional validation.

### Bayesian colocalization analysis

To identify genomic loci where biomarker-associated variants, tissue-specific eQTL signals, and gout risk variants share a single causal variant, we performed multi-trait Bayesian colocalization analysis using the HyPrColoc (Hypothesis Prioritization for multi-trait Colocalization) framework. Unlike pairwise methods, this algorithm allows for the simultaneous assessment of colocalization across multiple traits by clustering them based on shared genetic drivers. We evaluated the posterior probability (PP) that the biomarker GWAS, tissue-specific eQTL, and gout meta-GWAS define a shared cluster of localized association. We set a stringent threshold of PP > 0.7 to indicate significant evidence of multi-trait colocalization. This integrative approach requires concordance at the variant level across all three molecular data types, substantially reducing the likelihood of spurious associations driven by linkage disequilibrium.

### Functional gene validation

Candidate genes identified through both SMR analysis and colocalization were subjected to functional validation using quantitative reverse transcription PCR (qRT-PCR) and Western blot analysis in relevant tissue samples. For qRT-PCR, kidney tissue RNA was extracted using TRIzol reagent, and cDNA synthesis was performed using standard protocols. Real-time qRT-PCR was conducted in triplicate using PowerUp SYBR Green Master Mix, with thermal cycling optimized for each target. Relative gene expression was calculated using the 2^(-ΔΔCt) method normalized to housekeeping genes (GAPDH and ACTB). For Western blot analysis, total protein was extracted from kidney tissue lysates using RIPA buffer supplemented with protease and phosphatase inhibitors. Equal amounts of protein (30-50 μg) were resolved on polyacrylamide gels, transferred to PVDF membranes, and incubated with primary antibodies targeting candidate gene products. Protein bands were visualized using enhanced chemiluminescence and quantified by densitometry relative to β-actin or α-tubulin loading controls.

### Cell culture and treatment

THP-1 cells (ATCC TIB-202) were maintained in RPMI 1640 medium (Gibco) supplemented with 10% fetal bovine serum and 1% penicillin-streptomycin at 37 °C in 5% CO_2_. For macrophage differentiation, THP-1 cells were seeded at 2 × 10^5^ cells/ml and treated with phorbol 12-myristate 13-acetate (PMA; 100 ng/ml) for 48 hours. Following differentiation, cells were rested in PMA-free medium for 24 hours prior to treatment. Differentiated THP-1 macrophages were then stimulated with monosodium urate (MSU) crystals at 200 μg/ml for 24 hours, with PBS-treated cells serving as vehicle controls. All experiments were conducted in triplicate with a minimum of three independent biological replicates.

### RNA extraction and quantitative real-time PCR

To validate the transcriptional changes of the identified risk genes, total RNA was extracted from THP-1 macrophages treated with MSU crystals or vehicle control using TRIzol reagent (Invitrogen, USA) according to the manufacturer’s protocol. RNA concentration and purity were assessed using a NanoDrop 2000 spectrophotometer (Thermo Fisher Scientific), with acceptable samples having A_260_/_280_ ratios between 1.8 and 2.0. Subsequently, 1 μg of total RNA was reverse-transcribed into cDNA using the PrimeScript RT Reagent Kit (Takara, Japan).

Quantitative real-time PCR was performed using SYBR Green Master Mix (Applied Biosystems) on an ABI 7500 Real-Time PCR System with the following thermal cycling conditions: initial denaturation at 95 °C for 10 minutes, followed by 40 cycles of 95 °C for 15 seconds and 60 °C for 60 seconds. Target genes assessed included five protein-coding genes—*PRELID1*, *NIPAL1*, *LMAN2*, and *CAD*—and one long non-coding RNA (lncRNA), *AC093690*.1. GAPDH and ACTB were employed as reference genes for normalization. Relative expression levels were calculated using the comparative *2*^(–ΔΔ^*^Ct^*^)^ method and expressed as fold-change relative to vehicle-treated control cells. All primer sequences are listed in **Table 1**.

**Table 1:** Summary of prioritized effector genes identified through integrated SMR and colocalization analysis.

Gene	Tissue	β_SMR	P_SMR	P_HEIDI	PP_Coloc	Direction	Lead SNP	Associated metabolite
*PRELID1*	Kidney cortex	0.050	0.000297	0.458	**0.955**	**Risk**	rs6885410	Phospholipids in small VLDL
*LMAN2*	Whole blood	0.339	0.0000165	0.805	**0.945**	**Risk**	rs34604271	Triglycerides to total lipids ratio in very small VLDL
*NIPAL1*	Liver	0.074	0.000534	0.461	0.886	**Risk**	rs13146880	Triglycerides to total lipids ratio in very small VLDL
*CAD*	Whole blood	-0.340	0.0015	0.231	0.795	**Protective**	rs6547692	Triglycerides to total lipids ratio in very small VLDL
*ARL6IP5*	Liver	-0.060	0.00299	0.058	0.768	**Protective**	rs1965132	Phospholipid levels in chylomicrons and extremely large VLDL

Bold values and highlighted text indicate statistically significant associations, where the SMR P-value (P_SMR) is less than 0.05 and the HEIDI P-value (P_HEIDI) is greater than 0.05.

### Western blot analysis

To verify the protein abundance of the candidate genes, total protein was extracted from THP-1 cells using RIPA lysis buffer (150 mM NaCl, 1% Triton X-100, 0.5% sodium deoxycholate, 0.1% SDS, 50 mM Tris-HCl pH 8.0) supplemented with protease and phosphatase inhibitor cocktails (Roche). Protein concentration was determined using the BCA Protein Assay Kit (Pierce Biotechnology).

Given the wide range of molecular weights among the target proteins (25 kDa to 240 kDa), appropriately-sized SDS-PAGE gels were selected for each target: 6% polyacrylamide gels for the high-molecular-weight protein CAD (240 kDa), and 10-12% gels for PRELID1 (25 kDa), NIPAL1 (34 kDa), and LMAN2 (35 kDa). Protein samples (30-50 μg per lane) were separated under denaturing conditions and transferred onto PVDF membranes (Millipore) using a wet transfer system.

Membranes were blocked with 5% non-fat milk in Tris-buffered saline with 0.1% Tween 20 (TBST) for 1 hour at room temperature and incubated overnight at 4 °C with primary antibodies against PRELID1 (1:1000; Proteintech, 10877-1-AP), NIPAL1 (1:1000; Sigma-Aldrich, HPA036765), LMAN2 (1:1000; Proteintech; 11496-1-AP), and CAD (1:1000; Proteintech; 16617-1-AP). β-actin (1:5000; Proteintech; 20536-1-AP) was used as the loading control. Following three 10-minute washes in TBST, membranes were incubated with HRP-conjugated secondary antibodies (1:5000) for 1 hour at room temperature. Protein bands were visualized using enhanced chemiluminescence (ECL) reagent and quantified using ImageJ software. Band intensities for each target protein were normalized to the corresponding β-actin band intensity on the same membrane, and results are expressed as fold-change relative to vehicle-treated control cells.

### Software and analytical parameters

All statistical analyses were performed using R version 4.3.1 (R Foundation for Statistical Computing). Mendelian randomization analyses utilized the TwoSampleMR package (version 0.5.7) and Mendelian Randomization package (version 0.7.0). Instrumental variables were selected using genome-wide significance threshold (P < 5×10^-8^), followed by LD clumping (r² < 0.001 within 10,000 kb window) using PLINK v1.90 with the 1000 Genomes Phase 3 European reference panel. Instrument strength was verified using F-statistic > 10. Summary-data-based Mendelian randomization was performed using SMR software version 1.3.1. HEIDI test was applied with default parameters to distinguish causal associations from linkage. Bayesian colocalization analysis was performed using the HyPrColoc package (version 1.0.0) with default prior probabilities. GWAS meta-analysis was conducted using METAL software with inverse-variance weighted fixed-effects model. Heterogeneity across studies was assessed using Cochran’s Q statistic and I² index. All visualization was performed using ggplot2 (version 3.4.4) and ComplexHeatmap (version 2.16.0) packages.

## Results

### Meta-analysis of gout GWAS unveils robust genetic architecture

To establish a high-powered genetic foundation for identifying causal biomarkers, we performed a fixed-effects inverse-variance weighted meta-analysis of three large-scale gout GWAS datasets (N = 1,538,494). The final harmonized dataset covered 12,754,587 variants. We observed exceptional biological validity through the robust replication of established gout risk loci. Specifically, the classic missense variant in *ABCG2* (rs2231142) and the lead variant in *SLC2A9* (rs16890979) demonstrated associations exceeding genome-wide significance by orders of magnitude (*P* = 1.66×10^−305^ and *P* = 1.04×10^−305^, respectively), confirming the directionality and phenotype specificity of our meta-analysis. Furthermore, the genomic inflation factor (*λGC*) was 1.08, indicating that population stratification was effectively controlled and that the observed signals are driven by genuine polygenic architecture rather than systemic bias. We identified statistically significant heterogeneity in a subset of variants, which were rigorously excluded from downstream instrumental variable selection to ensure the robustness of the MR analyses. The resulting high-quality summary statistics served as the primary outcome for the subsequent multi-omics causal inference.

### Metabolomic associations with gout

**Figure 1:**
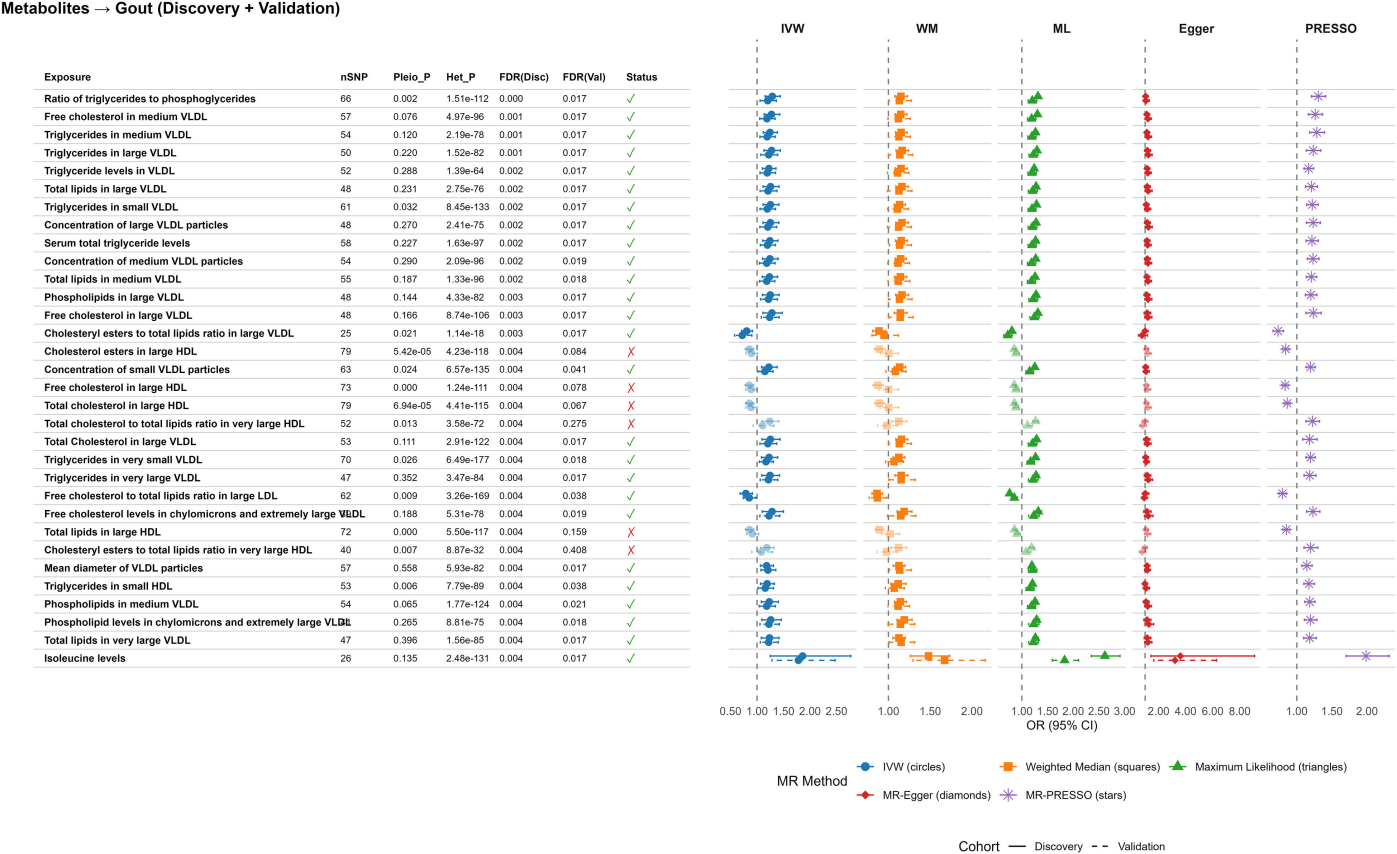
Causal associations of circulating metabolic biomarkers with gout risk. Forest plot displaying the Mendelian Randomization (MR) estimates for circulating metabolites significantly associated with gout. Results are shown for both the discovery phase and the replication phase. The x-axis represents the odds ratio (OR) per standard deviation increase in the genetically predicted metabolite level; error bars indicate 95% confidence intervals. Colors denote different MR methods (Blue: Inverse Variance Weighted [IVW]; Orange: Weighted Median; Green: Maximum Likelihood; Red: MR-Egger). All associations shown passed FDR correction (*P_FDR_* < 0.05) in the discovery phase. Different MR methods are distinguished by both shape and color: IVW (circles), Weighted Median (squares), Maximum Likelihood (triangles), MR-Egger (diamonds).

In MR analyses of 233 circulating metabolites, 32 showed significant causal associations with gout after FDR correction (*P_FDR_* < 0.05), and all 32 replicated in FinnGen with consistent direction of effect (nominal *P* < 0.05). Most signals were lipid-related and clustered in triglyceride-rich very-low-density lipoprotein (VLDL) particles. Triglycerides and cholesterol components in medium and large VLDL were consistently associated with higher gout risk (e.g., triglycerides in medium VLDL: OR_discovery_ = 1.24, 95% CI 1.12–1.38; OR_validation_ = 1.19, 95% CI 1.05–1.34), and both VLDL particle concentrations and mean VLDL diameter were positively associated with gout. Similar patterns were observed for chylomicrons and extremely large VLDL, extending the lipoprotein–gout association across multiple particle classes (**Figure 1**).

Amino acid profiling identified circulating isoleucine as a strong causal risk factor, with larger effect sizes than lipid traits (OR_discovery_ = 1.86, 95% CI 1.25–2.77; OR_validation_ = 1.78, 95% CI 1.28–2.47), robust across multiple MR methods. In contrast, several lipoprotein compositional ratios showed protective associations, including the phospholipids-to-total lipids ratio in medium VLDL (OR_discovery_ = 0.86, 95% CI 0.78–0.96) and the free cholesterol-to-total lipids ratio in large HDL (OR_discovery_ = 0.84, 95% CI 0.73–0.96), suggesting that specific features of lipoprotein composition may mitigate gout risk (**Figure 1**).

### Lipidomic associations with gout

**Figure 2:**
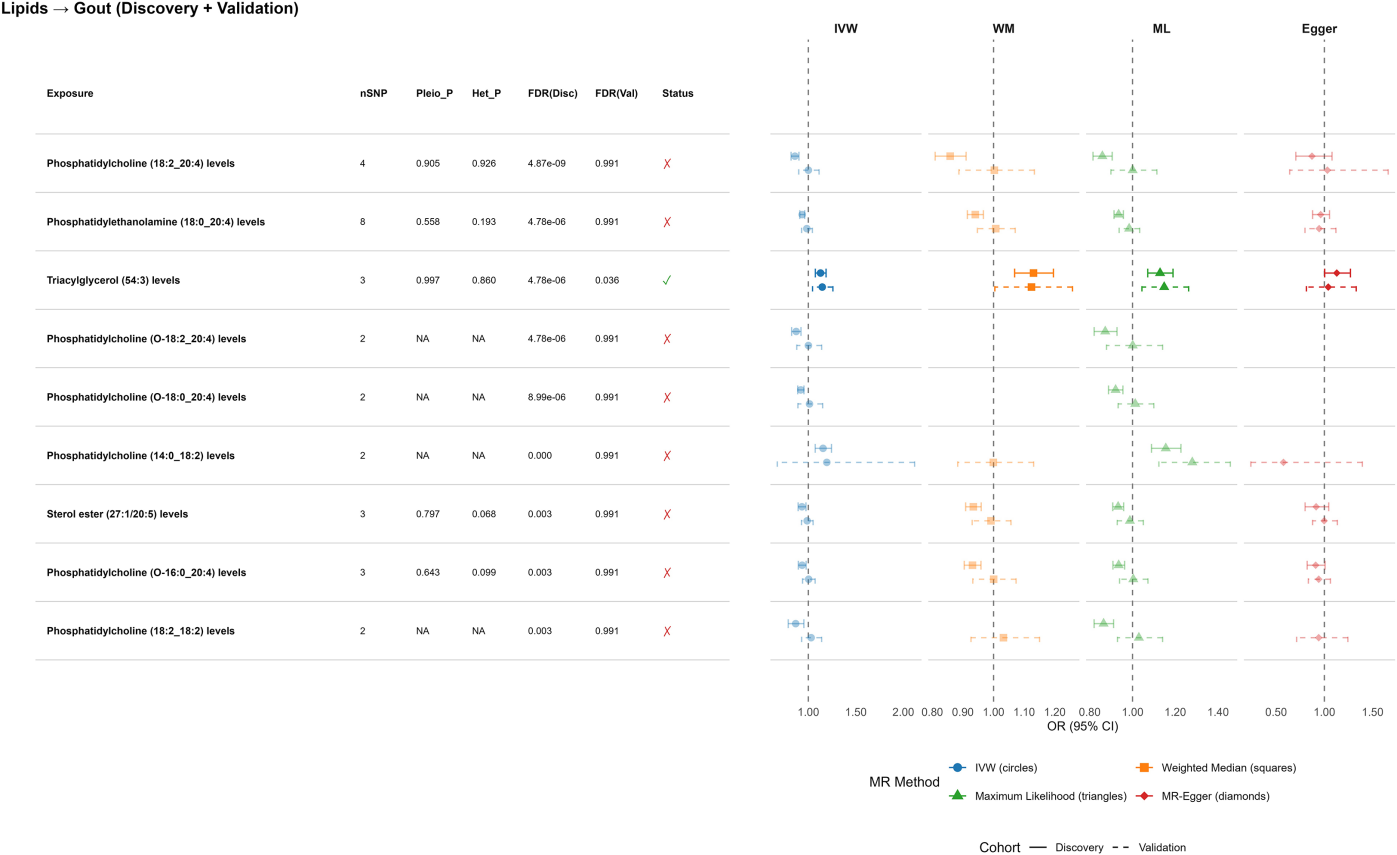
Mendelian randomization analysis of plasma lipid species and gout susceptibility. Forest plot illustrating the causal effect estimates for plasma lipid species on gout risk. The plot compares the primary IVW method with sensitivity analyses (Weighted Median, Maximum Likelihood, MR-Egger) to demonstrate the robustness of the association. Points represent the odds ratio (OR), and horizontal lines represent 95% confidence intervals. Different MR methods are distinguished by both shape and color: IVW (circles), Weighted Median (squares), Maximum Likelihood (triangles), MR-Egger (diamonds).

Among 179 lipid species, multiple showed suggestive associations, but only triacylglycerol TAG (54:3) remained significant after FDR correction and successfully replicated in the independent cohort (OR_discovery_ = 1.13, 95% CI 1.07–1.19, FDR = 6.33×10^-5^; OR_validation_ = 1.15, 95% CI 1.04–1.26). Sensitivity analyses and pleiotropy tests supported a causal effect. TAG (54:3), a marker of triglyceride-rich lipoproteins, is consistent with and likely reflects the broader VLDL-related metabolomic signals, while the overall lipidomic signal landscape remained sparse (**Figure 2**).

### Proteomic associations with gout

**Figure 3:**
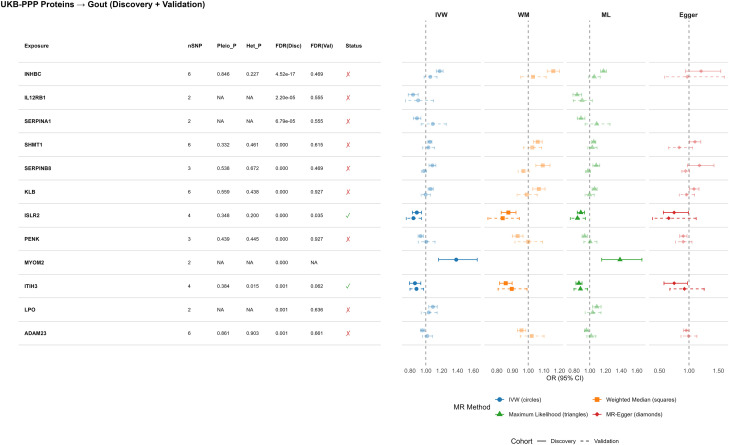
Proteome-wide Mendelian randomization identifies causal plasma protein regulators of gout. Causal estimates for plasma proteins identified as significant determinants of gout risk. The analysis integrates proteomic data from the UK Biobank Pharma Proteomics Project (UKB-PPP) and the deCODE consortium. The forest plot displays the odds ratio (OR) for gout per unit increase in normalized protein expression levels. Consistency across different MR estimators (IVW, Weighted Median, ML, Egger) supports the validity of these protective associations. Different MR methods are distinguished by both shape and color: IVW (circles), Weighted Median (squares), Maximum Likelihood (triangles), MR-Egger (diamonds).

Proteome-wide MR of 926 plasma proteins identified two proteins with FDR-significant causal associations with gout, both protective: ISLR2 and ITIH3. Higher ISLR2 levels were associated with lower gout risk (OR_discovery_ = 0.89, 95% CI 0.83–0.95; OR_validation_ = 0.84, 95% CI 0.75–0.94), as were higher ITIH3 levels (OR_discovery_ = 0.86, 95% CI 0.79–0.94; OR_validation_ = 0.88, 95% CI 0.80–0.97). These associations were robust across alternative MR estimators, and pleiotropy/heterogeneity tests did not indicate major violations of MR assumptions, supporting a protective role of ISLR2 and ITIH3 in gout pathogenesis (**Figure 3**). Pleiotropy assessment for both proteins revealed minimal violations of MR assumptions.

### Summary-data-based Mendelian randomization and gene mapping

**Figure 4:**
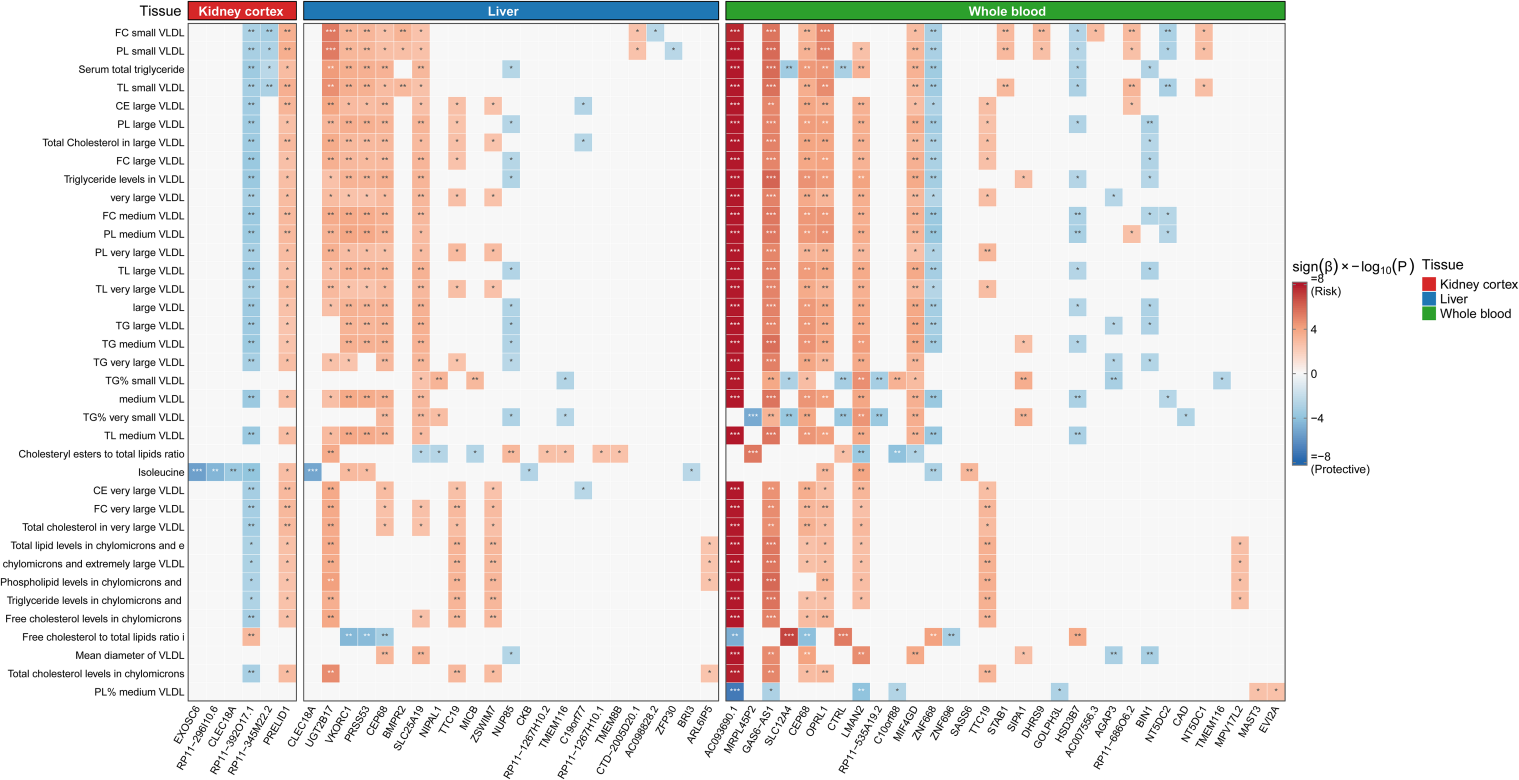
Summary-data-based Mendelian randomization (SMR) heatmap integrating GWAS and eQTL data across three tissues: Kidney Cortex, Liver, and Whole Blood. Color scale represents direction-weighted significance: blue indicates protective associations (negative β, higher expression associated with decreased gout risk), red indicates risk associations (positive β, higher expression associated with increased gout risk). Color intensity corresponds to sign(β_SMR) × -log_10_(P_SMR). Asterisks indicate significance levels: *P < 0.01, **P < 0.001, ***P < 0.00001.

To elucidate the transcriptomic architecture underlying the identified metabolic associations, we performed a multi-tissue SMR analysis, summarizing the prioritized effector genes and their tissue-specific expression patterns in **Figure 4**. The kidney cortex emerged as a key regulatory tissue, containing genes with distinct risk-increasing and protective profiles. *PRELID1* (prelipin domain–containing 1) was identified as a significant renal risk gene that higher cortical expression was causally associated with increased gout risk (β_SMR_ = 0.050, *P_SMR_* = 9.22×10^-5^, *P_HEIDI_* = 0.46) and with elevated levels of isoleucine and cholesteryl esters in large VLDL (β_SMR_ = 0.030, *P_SMR_* = 2.97×10^-4^), supporting a specific amino acid– and lipid-driven risk pathway. By contrast, we observed robust protective mechanisms in the kidney. *CLEC18A* (C-type lectin domain containing 18A) showed a significant inverse association with gout, whereby higher expression was linked to reduced disease susceptibility (β_SMR_ = −0.078, *P_SMR_* = 1.95×10^-5^, *P_HEIDI_* = 0.50) and to lower circulating isoleucine levels. In addition, the long non-coding RNA *RP11-392O17.1* displayed a protective profile that lower expression was causally associated with reduced isoleucine abundance (β_SMR_ = −0.032, *P_SMR_* = 1.11×10^-4^) and decreased gout risk (β_SMR_ = −0.034, *P_SMR_* = 7.99×10^-4^), implicating renal non-coding regulatory elements in metabolic pathogenesis.

Hepatic transcriptomic signals showed extensive colocalization with lipid-rich metabolic traits. *NIPAL1* (NIPA-like domain containing 1) emerged as the top hepatic risk gene, with higher liver expression exerting a strong causal effect on gout risk (β_SMR_ = 0.074, *P_SMR_* = 1.52×10^-8^, *P_HEIDI_* = 0.46) and on total lipids in chylomicrons and extremely large VLDL. The glucuronosyltransferase *UGT2B17* provided particularly compelling evidence for a coordinated gene–metabolite–disease axis. Increased hepatic *UGT2B17* expression was associated with both higher gout risk (β_SMR_ = 0.040, *P_SMR_* = 3.96×^-5^) and increased levels of serum total triglycerides and triglycerides in large VLDL (β_SMR_ = 0.038, *P_SMR_* = 3.91×10^-6^). This concordant directionality suggests that enhanced hepatic glucuronidation capacity may pathologically influence the abundance of triglyceride-rich lipoproteins, thereby predisposing individuals to gout.

In whole blood, we identified strong associations reflecting systemic metabolic regulation. The lncRNA *AC093690.1* showed one of the largest effects in the study that higher expression was causally linked to a marked increase in gout risk (β_SMR_ = 0.155, *P_SMR_* = 1.03×10^-9^, *P_HEIDI_* = 0.11) and was strongly associated with elevated serum isoleucine levels (β*_SMR_* = 0.106, *P_SMR_* = 1.77×10^-10^). Additionally, *LMAN2* (lectin, mannose-binding 2) was identified as a substantial risk factor (β_SMR_ = 0.339, *P_SMR_* = 0.002), with its expression specifically linked to the mean diameter of VLDL particles, further supporting the systemic contribution of glycoprotein transport pathways to lipoprotein remodeling and disease etiology.

### Bayesian colocalization analysis

To definitively establish that the identified eQTLs, metabolic biomarkers, and gout risk are driven by shared genetic variants rather than coincidental overlap due to linkage disequilibrium, we performed multi-trait Bayesian colocalization analysis using the HyPrColoc framework. Strong evidence of colocalization was observed for our top prioritized candidate genes, reinforcing their roles as specific effectors of metabolic risk pathways. In the kidney cortex, we detected a robust colocalization signal at the *PRELID1* locus (PP = 0.955). The variant rs6885410 was identified as the shared causal driver linking renal *PRELID1* expression, alterations in phospholipid levels in small VLDL particles, and gout susceptibility, confirming *PRELID1* as a high-confidence renal effector gene. In whole blood, *LMAN2* exhibited extremely strong colocalization (PP = 0.945), with rs34604271 serving as the shared variant influencing the triglycerides-to-total-lipids ratio in very small VLDL. Similarly, *CAD* showed substantial evidence of colocalization (PP = 0.795) mediated by rs6547692, validating the mechanistic link between pyrimidine/purine metabolism, VLDL composition, and gout risk. Hepatic regulation was highlighted by *NIPAL1*, which demonstrated a high posterior probability of 0.886 (candidate SNP rs13146880) in the liver, linking hepatic expression to VLDL triglyceride ratios.

### Experimental validation of prioritized risk genes

**Figure 5:**
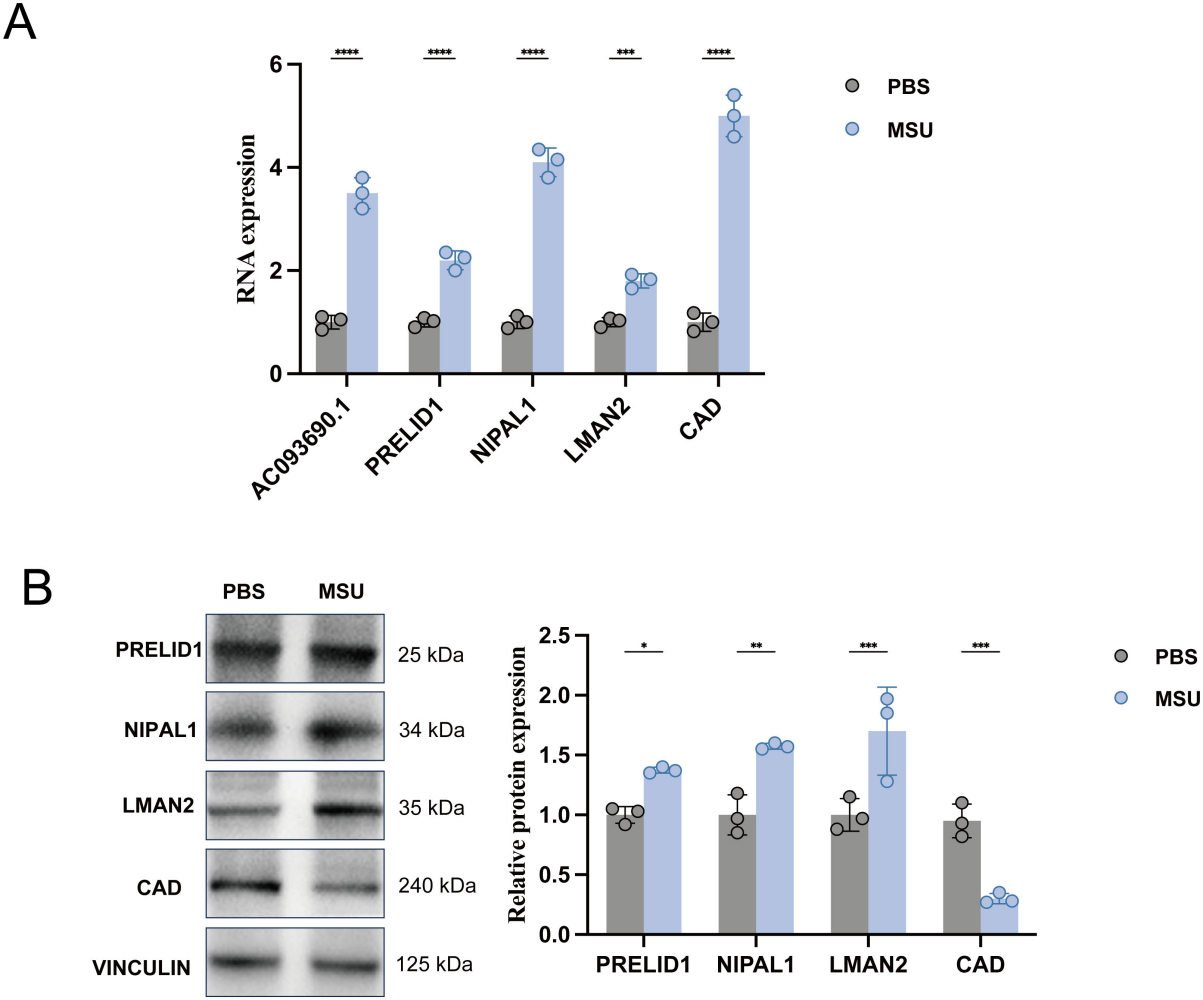
Experimental validation of prioritized effector genes in an *in vitro* gout model. **(A)** Quantitative Real-Time PCR (qPCR) analysis of mRNA expression levels for *PRELID1*, *NIPAL1*, *LMAN2*, *CAD*, and the lncRNA *AC093690.1* in PMA-differentiated THP-1 macrophages. Cells were stimulated with Monosodium Urate (MSU) crystals (200 *μ*g/mL) or vehicle control (PBS) for 24 hours. Data are expressed as fold change relative to the control group (normalized to *GAPDH* and *ACTB*). Error bars represent standard deviation (SD). *P < 0.05 *vs*. PBS (Vehicle). **(B)** Western blot analysis and densitometric quantification of protein abundance for PRELID1 (25 kDa), NIPAL1 (34 kDa), LMAN2 (35 kDa), and CAD (240 kDa) in THP-1 cell lysates following MSU stimulation. *β*-actin was used as a loading control. Consistent with the mRNA and SMR results, protein levels of risk genes (*PRELID1, NIPAL1, LMAN2*) were upregulated, while the protective factor *CAD* was downregulated under inflammatory conditions. *P < 0.05, **P 0.01, ***P 0.001, and ****P 0.0001 versus the PBS control group.

To verify the biological relevance of our computationally predicted effector genes, we assessed their transcriptional and translational responses in an *in vitro* model of acute gouty inflammation. Differentiated THP-1 macrophages were stimulated with MSU crystals (200 *μ*g/mL), mimicking the pathogenic microenvironment of gout. qPCR analysis (**Figure 5A**) revealed significant transcriptional dysregulation among all five prioritized candidates upon MSU stimulation. Consistent with their identification as risk genes in our SMR analysis, the mRNA expression levels of *PRELID1*, *NIPAL1*, and *LMAN2* were significantly upregulated in the MSU-treated group compared with vehicle controls (*P* < 0.05). Similarly, the long non-coding RNA *AC093690.1* exhibited a marked increase in expression, validating its putative role as a pro-inflammatory regulator. Conversely, *CAD*, which was identified as a protective factor in our SMR analysis, displayed a significant downregulation in response to MSU crystals, further supporting its potential role in mitigating gout susceptibility.

We next sought to confirm whether these transcriptional changes translated to alterations in protein abundance. Western blot analysis of THP-1 cell lysates demonstrated concordance with the mRNA findings (**Figure 5B**). The protein levels of PRELID1, NIPAL1, and LMAN2 were substantially elevated following MSU exposure. In parallel, CAD protein abundance was significantly reduced in the inflammatory state. These experimental data provide crucial biological validation of our multi-omics framework, demonstrating that these genetically causal genes are dynamically regulated during the acute inflammatory response characteristic of gout pathogenesis.

## Discussion

This study represents the most comprehensive multi-omics dissection of gout etiology to date, triangulating evidence across genomic, transcriptomic, metabolomic, and proteomic layers to elucidate the causal hierarchy of disease. By integrating a high-power meta-GWAS (N>1.5 million) with tissue-specific SMR and experimental validation, we moved beyond simple association to identify specific molecular effectors. Our findings fundamentally reframe gout not merely as a disorder of renal urate under-excretion, but as a systemic metabolic pathology driven by specific lipoprotein dysregulation, amino acid imbalances, and mitochondrial-immune cross-talk.

A salient finding of our work is the causal implication of specific lipid species and lipoprotein characteristics—particularly VLDL particle size and triglyceride content—in gout etiology. While hypertriglyceridemia is a known comorbidity of gout, our MR analyses refine this association, pinpointing large VLDL particles and circulating isoleucine as independent causal drivers. Mechanistically, we mapped these associations to *NIPAL1* in the liver and *LMAN2* in whole blood. *NIPAL1*, which encodes a magnesium transporter, was identified as a risk gene linked to lipid abundance. Recent evidence suggests that *NIPAL1* dysfunction compromises insulin secretion and magnesium homeostasis, key features of the metabolic syndrome often antecedent to gout (17, 18). We propose that *NIPAL1*-mediated perturbations in hepatic divalent cation balance may impair insulin signaling, thereby promoting hepatic VLDL overproduction and subsequent hyperuricemia through shared renal secretory competition. Furthermore, the identification of *LMAN2* as a causal risk factor linked to VLDL diameter offers a novel intersection between glycoprotein transport and lipid metabolism. *LMAN2* facilitates the sorting of glycosylated cargo in the secretory pathway; its upregulation may enhance the secretion of pro-atherogenic, triglyceride-rich lipoproteins or inflammatory cytokines, providing a molecular basis for the well-documented “inflammatory lipid” phenotype in gout patients. It is important to note that our MR analysis used clinically diagnosed gout as the outcome, which encompasses both hyperuricemic predisposition and inflammatory response to MSU crystals. The VLDL-triglyceride associations may therefore operate through dual mechanisms: a metabolic pathway whereby triglyceride-rich lipoproteins compete with uric acid for renal organic anion transporters, impairing urate clearance; and an inflammatory pathway whereby triglyceride-enriched lipoproteins prime macrophages for enhanced NLRP3 inflammasome activation upon MSU exposure (19).

Our analysis also highlights a critical, underappreciated role for renal mitochondrial integrity in urate homeostasis through the identification of *PRELID1*. This gene, which facilitates the transfer of phosphatidic acid across the mitochondrial intermembrane space, was a strong causal risk factor in the kidney cortex. The proximal tubule, the primary site of urate reabsorption and secretion, is highly metabolically active and dependent on mitochondrial ATP generation to power transporters such as ABCG2 and URAT1. We hypothesize that overexpression of *PRELID1* leads to mitochondrial lipid imbalance and dysfunction, as observed in other models of renal injury (20). This bioenergetic deficit may selectively impair the high-energy demands of urate excretion, leading to hyperuricemia. The upregulation of *PRELID1* in our MSU-stimulated macrophage model further suggests that this mitochondrial stress response is not only a predisposing factor but is exacerbated during acute inflammation, potentially creating a vicious cycle of metabolic stress and NLRP3 inflammasome activation.

We also found that genetically higher expression of *CAD* reduces gout risk, while acute inflammation (MSU stimulation) suppresses its expression. This inverse relationship points to a competitive interplay between purine and pyrimidine metabolic pools. Biologically, purine and pyrimidine synthesis compete for the shared substrate phosphoribosyl pyrophosphate (PRPP). Reduced *CAD* function would theoretically spare PRPP, shunting it towards purine synthesis and increasing uric acid production. However, our findings suggest a more complex dynamic, potentially involving the salvage pathway or immune-metabolic regulation, where CAD downregulation serves as a compensatory brake on inflammation. To clarify this inverse relationship: individuals with higher baseline CAD expression have lower gout risk because CAD-mediated pyrimidine synthesis diverts PRPP away from purine synthesis, thereby limiting uric acid production. During acute MSU-induced inflammation, the observed CAD downregulation represents a pathological metabolic shift that removes this protective brake, creating a feed-forward inflammatory cycle (21). The suppression of *CAD* by MSU crystals observed in our functional validation suggests that acute inflammation may shift metabolic flux toward purine catabolism, perpetuating the hyperuricemic state.

The strengths of this study lie in its integrative design and dual validation strategy. By combining statistical triangulation (MR, SMR, Colocalization) with biological validation in human macrophages, we minimized the false-positive rates inherent in pure “in silico” studies. However, limitations exist. First, our analyses were restricted to European ancestry populations, potentially limiting generalizability to other ethnic groups. Second, the *in vitro* validation using THP-1 cells, while informative, does not fully recapitulate the complexity of *in vivo* gouty inflammation involving multiple cell types and tissue compartments. Third, the cross-sectional nature of GWAS data precludes assessment of temporal dynamics between biomarker changes and disease onset. Fourth, some identified genes lack well-characterized biological functions, necessitating further mechanistic investigation. Fifth, while our SMR analysis identified tissue-specific effector genes in the kidney cortex (PRELID1) and liver (NIPAL1), experimental validation was performed exclusively in THP-1 macrophages. Although these genes are expressed in monocyte-derived cells and macrophages are central to MSU-induced inflammation, this model cannot fully recapitulate the tissue-specific physiological context. Future studies utilizing primary renal tubular epithelial cells and hepatocytes will be essential to confirm the causal roles of these genes in their respective tissues. Fifth, while our SMR analysis identified tissue-specific effector genes in the kidney cortex (PRELID1) and liver (NIPAL1), experimental validation was performed exclusively in THP-1 macrophages. Although these genes are expressed in monocyte-derived cells and macrophages are central to MSU-induced inflammation, this model cannot fully recapitulate the tissue-specific physiological context. Future studies utilizing primary renal tubular epithelial cells and hepatocytes will be essential to confirm the causal roles of these genes in their respective tissues.

## Conclusion

In summary, this study unveils a high-resolution molecular map of gout pathogenesis, identifying *PRELID1*, *NIPAL1*, and *LMAN2* as novel, causally validated effector genes. We establish that gout risk is actively modulated by upstream regulators of VLDL assembly and mitochondrial integrity, rather than passive urate handling alone. The discovery of protective proteins (*ISLR2*, *ITIH3*) and specific metabolic drivers (*Isoleucine*) broadens the therapeutic armamentarium beyond xanthine oxidase inhibitors. These findings advocate for a precision medicine approach to gout management, targeting specific metabolic and inflammatory nodes to arrest disease progression.

## Data Availability

The original contributions presented in the study are included in the article/**Supplementary Material**. Further inquiries can be directed to the corresponding author.
